# Supplementary material for: Composition and acquisition of the microbiome in solitary, ground-nesting alkali bees
Source: Sci Rep. 2021 Feb 4;11:2993. doi: 10.1038/s41598-021-82573-x (PMC7862682; doi:10.1038/s41598-021-82573-x)
Supplement: Supplementary file 2 — Supplementary Legends. [file 41598_2021_82573_MOESM2_ESM.docx]

**SUPPLEMENTAL TABLE LEGENDS**

**Table S1.** Taxa (ASVs) with significantly differential abundance among adult females. ‘Contrast’ is the comparison in question; ‘ASV’ ASV identification number; ‘baseMean’ Average read count; ‘log2FoldChange’ log2 based difference between the two groups in the contrast; ‘lfcSE’ standard error of the log fold change; ‘stat’ test statistic; ‘pvalue’ p-value; ‘BH-adjusted pvalue’ Benajmini-Hochberg adjusted p-value; ‘Kingdom’, ‘Phylum’, ‘Class’, ‘Order’, ‘Family’, ‘Genus’, ‘Species’ all correspond to the classification of each ASV.

**Table S2.** Taxa (ASVs) classified as *Lactobacillus micheneri*. ‘fig5_label’ is the ASV label in Fig. 5; ‘OTU’ is the ASV identification number; ‘sample_type’ is the group to which each summary statistic is applied; ‘mean’ is the average read count for each ASV within each sample type; ‘sd’ standard deviation; ‘N’ sample size; ‘min’ minimum read count’; ‘max’ maximum read count
